# Structure‐Foldable and Performance‐Tailorable PI Paper‐Based Triboelectric Nanogenerators Processed and Controlled by Laser‐Induced Graphene

**DOI:** 10.1002/advs.202310017

**Published:** 2024-05-15

**Authors:** Weixiong Yang, Mingguang Han, Fu Liu, Dan Wang, Yan Gao, Guantao Wang, Xilun Ding, Sida Luo

**Affiliations:** ^1^ School of Mechanical Engineering & Automation Beihang University No. 37 Xueyuan Road Beijing 100191 China; ^2^ Shenzhen Institute of Beihang University No. 51 GaoxinSouth 9th Road Guangdong 518063 China

**Keywords:** energy harvesting, laser‐induced graphene, paper electronics, tactile sensors, triboelectric nanogenerators

## Abstract

Laser‐induced graphene (LIG) technology has provided a new manufacturing strategy for the rapid and scalable assembling of triboelectric nanogenerators (TENG). However, current LIG‐based TENG commonly rely on polymer films, e.g., polyimide (PI) as both friction material and carbon precursor of electrodes, which limit the structural diversity and performance escalation due to its incapability of folding and creasing. Using specialized PI paper composed of randomly distributed PI fibers to substantially enhance its foldability, this work creates a new type of TENG, which are structurally foldable and stackable, and performance tailorable. First, by systematically investigating the laser power‐regulated performance of single‐unit TENG, the open‐circuit voltage can be effectively improved. By further exploiting the folding process, multiple TENG units can be assembled together to form multi‐layered structures to continuously expand the open‐circuit voltage from 5.3 to 34.4 V cm^−2^, as the increase of friction units from 1 to 16. Last, by fully utilizing the unique structure and performance, representative energy‐harvesting and smart‐sensing applications are demonstrated, including a smart shoe to recognize running motions and power LEDs, a smart leaf to power a thermometer by wind, a matrix sensor to recognize writing trajectories, as well as a smart glove to recognize different objects.

## Graphical abstract

Using laser‐induced graphene (LIG) technology and polyimide (PI) paper, this work describes a foldable and tailorable PI paper‐based triboelectric nanogenerator (PIP‐TENG). The laser with variable processing parameters can rapidly regulate and produce high‐performance PIP‐TENG with graphene electrodes. Accordingly, the PIP‐TENG with desirable electrical energy output can be applied to collect wind energy and sense human motions.

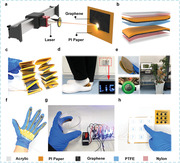

## 1 Introduction

In recent years, triboelectric nanogenerators (TENG) have rapidly been developed into a hot topic in the fields of sustainable energy utilization and industrial sensing due to their unique working principle and broad application prospects.^[^ ^1^, ^2^ ^]^ As a new type of energy‐harvesting device, TENG invented by Wang's group^[^ ^3^ ^]^ could motivate electrical energy by coupled contact electrification and electrostatic induction to convert tiny energy into a stable energy supply.^[^ ^4^, ^5^, ^6^, ^7^, ^8^ ^]^ Due to the unique merits of high flexibility, simple structure, low production cost, and material diversity, a lot of TENG‐based smart devices have been developed for energy harvesting applications, such as natural energy collectors,^[^ ^9^, ^10^, ^11^, ^12^ ^]^ micro‐supercapacitors,^[^ ^13^, ^14^ ^]^ smart wearables,^[^ ^15^, ^16^, ^17^, ^18^ ^]^ human–computer interfaces,^[^ ^19^, ^20^, ^21^, ^22^, ^23^ ^]^ and intelligent sensors.^[^ ^24^, ^25^, ^26^, ^27^, ^28^ ^]^ Although traditional electrode materials of TENG such as metals, conducting polymers, hydrogels and ionic gels could play key roles in performance improvement,^[^ ^29^, ^30^ ^]^ the manufacturing process of coating, sputtering, inkjet printing and aerosol‐jet printing still fails to meet the requirement of flexible application scenario and device integration,^[^ ^31^, ^32^ ^]^ attributed to the shortcomings of cumbersome processing steps, strict processing conditions, high costs, difficult assembly, and single structure and function, etc. Accordingly, a brand‐new protocol for cost‐effective and highly efficient manufacturing of TENG electrodes is highly anticipated.

As an emerging technology, laser‐induced graphene (LIG) has exhibited multiple unique advantages including high fabrication efficiency, low cost, and designable structures owing to the one‐step laser‐scribing process to precisely and programmatically synthesize porous graphene electrodes from specific polymer films,^[^ ^33^, ^34^, ^35^ ^]^ which is crucial to assemble smart/functional devices applied in energy‐storing and wearable applications.^[^ ^36^, ^37^ ^]^ Therefore, multiple works have started applying LIG‐enabled electrodes in TENG due to their unique electrical conductivity, thereby improving production efficiency and simplifying TENG structures.^[^ ^38^, ^39^, ^40^, ^41^ ^]^ For example, James M. Tour was the pioneer to introduce LIG technology for assembling TENG in 2019^[^ ^42^ ^]^ and successfully proposed a highly performed LIG triboelectric nanogenerators (LIG‐TENG) through laser irradiation processed on a polyimide (PI) film.^[^ ^43^, ^44^ ^]^ Due to the urgent need for flexible microelectronic devices and wearable systems, flexible LIG‐TENG are widely concerned and thus enormous efforts have been devoted to film‐based composite electrodes by using polymer film precursors. In detail, LIG/polymer film‐based TENG could harvest energy via various motion modes such as folding, bending, and stretching to meet the requirements of various devices.^[^ ^45^, ^46^, ^47^, ^48^ ^]^ However, polymeric films with high elasticity and high yield strength are hard to occur dissipative force‐deformation behaviors, which are crucial to maintain folding states, thus leading to limited topological structures of TENG that may not be able to fulfill multi‐class wearable devices.^[^ ^49^ ^]^ Therefore, it is necessary to propose a facile and scalable process of LIG‐TENG with variously foldable structures.

Compared with polymer films, paper‐based polymers composed of randomly distributed fibers could exhibit superior flexibility and foldability. Since the nonwoven fiber network of polymer papers or mats performs higher stiffness and lower yield strength, various folding structures could be guaranteed because of higher dissipative behaviors.^[^ ^49^, ^50^ ^]^ Here, a simple and scalable LIG electrode fabrication strategy was proposed to produce PI paper‐based triboelectric nanogenerators (PIP‐TENG) with foldable structure and tailorable performance via precise control of critical laser parameters. As for performance adjustment, a series of PIP‐TENG with both single‐unit and multi‐unit structures were examined under various laser powers and defocus distances. Under laser power of 1.25 W and defocus distance of 0 mm, the single‐unit PIP‐TENG with optimized performance of electrical‐energy generation were achieved with the open‐circuit voltage of 430 V (corresponding to 11.9 V cm^−2^) and the peak power of 5 mW. To further extend the structural diversity as well as the energy generation performance, origami structures of PIP‐TENG with multi‐unit stacking number as high as 16 were designed to achieve a 6‐fold enhancement of open‐circuit voltage from 5.3 to 34.4 V cm^−2^ compared with a single‐unit device, demonstrating the unique advantage of the developed fabrication process. Notably, the optimized PIP‐TENG could also exhibit excellent structural durability by sustaining a long‐term impact operation of 6 h and 108000 cycles without altering its performance. To best utilize its performance, representative energy‐harvesting and smart‐sensing applications have been demonstrated. For demonstrating wearable applications with energy‐harvesting performance, a PIP‐TENG‐based smart shoe was developed to recognize walking and running motions and power 22 LEDs. For demonstrating bionic structures with energy‐harvesting performance, multiple PIP‐TENG‐based leaf‐shaped devices were fabricated to collect wind energy for driving various electronic devices including temperature and humidity meters as well as 60 LEDs. Lastly, for demonstrating wearable applications with tactile‐sensing performance, a PIP‐TENG‐based palm tactile sensor and a  matrix sensor were developed to accurately identify the contact and grasping movements of each finger and to monitor various writing gestures to control the screen. It is thus highly expected that the PI paper‐enabled PIP‐TENG technology can greatly increase production efficiency and extend multi‐scenario applications of smart sensors, wearable electronics, energy‐harvesting devices, and intelligent robots in the future.

## 2 Results and Discussion

### 2.1 Structure and Working Mechanism

**Figure 1:**
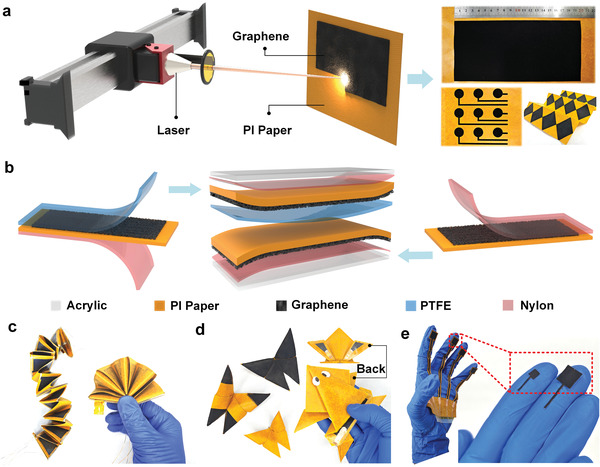
a) Schematic diagram of laser processing and pictures of LIG patterns on a PI paper substrate. b) Schematic diagram of multi‐layered structures of PIP‐TENG. c) Pictures of snake‐shaped and fan‐shaped origami PIP‐TENG. d) Pictures of butterfly‐shaped and frog‐shaped PIP‐TENG. e) Pictures of palm tactile sensors based on PIP‐TENG.

The processing of LIG and the assembly of TENG are shown in **Figure** **1**. Figure 1a shows the fabrication of a porous graphene network on top of the PI paper substrate via line‐by‐line scribing of laser beam. It has been demonstrated that LIG has remarkable electrical conductivity and can be used as electrodes for TENG.^[^ ^42^ ^]^ Due to patternable and programmable features of laser processing, various complex graphene electrode patterns could be fabricated, e.g., a large‐area rectangle, a 3 by 3 array of round disks, and 12 rhombuses. After producing LIG, the top of graphene layer is covered with polytetrafluoroethylene (PTFE) film to form the negative electrode and one friction interface of TENG (Figure 1b), while another pristine LIG layer functions as the positive electrode and another friction interface. Thus, the PTFE film of negative electrode and the remaining PI layer of positive electrode could contact with each other to form the friction pair. Lastly, the outer surface of both the positive and negative electrodes is covered with a nylon film, and an acrylic plate is overlaid on top of both the nylon films to form a load‐transferring layer. Overall, the applied LIG technology can process complex electrodes rapidly, precisely, and optionally, while endowing the PIP‐TENG with excellent folding properties and microsensing capabilities. For example, it can be fashioned into various origami and biomimetic strucutres, including a snake and a fan (Figure 1c), a butterfly and a frog (Figure 1d; Figure S12, Supporting Information), and multiple palm tactile sensors (Figure 1e). Expectedly, TENG produced by this LIG technology could be applied to environmental energy collection, smart wearable devices, and sensing and monitoring systems. Thus, TENG with different application requirements can be promised through this novel method by offering different structurally‐foldable configurations.

**Figure 2:**
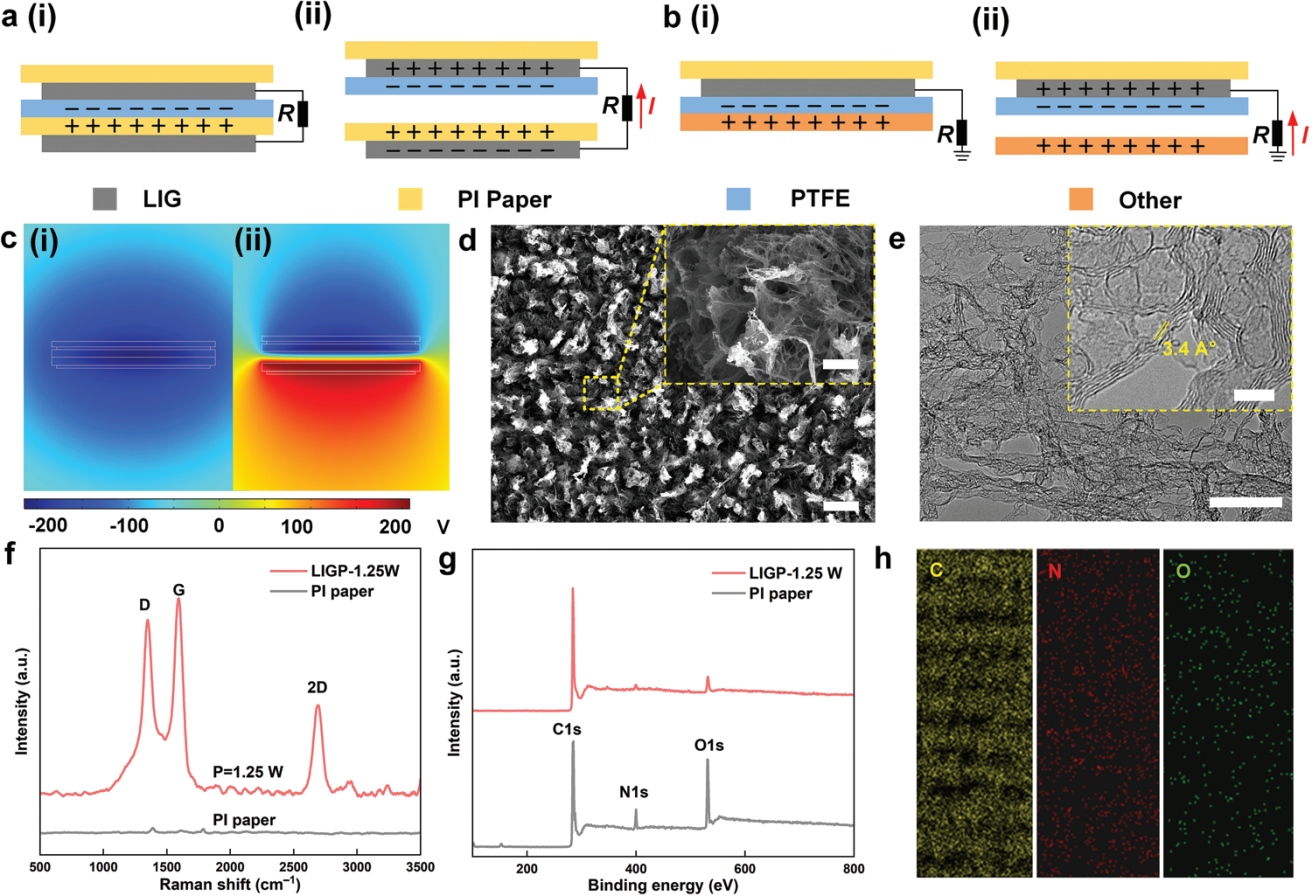
a) Operation principle of contact‐separation mode in TENG. b) Operation principle of single‐electrode mode in TENG. c) Potential simulation of contact‐separation mode in TENG. d) SEM images of LIG‐1.25 under low (scale = 100 µm) and high magnifications (inset, scale = 5 µm). e) TEM images of LIG‐1.25 under low (scale = 100 nm) and high magnifications (inset, scale = 10 nm). f) Comparison of Raman between LIG‐1.25 and pristine PI paper. g) XPS survey of pristine PI and LIG‐1.25. h) The EDS mapping of C, N, and O elements from LIG‐1.25.

**Figure** **2**a,b illustrate the operation principle of PIP‐TENG. In this study, two types of PIP‐TENG were fabricated, including a contact‐separation mode TENG (Figure 2a) and a single‐electrode TENG (Figure 2b). Due to frictional effects, an equal number of opposite charges will be generated on the surface of the PTFE and PI paper when the electrodes come into contact (Figure 2a i). Due to the principle of electrostatic induction, when the two electrodes are separated (Figure 2a ii), the graphene electrodes in the positive and negative electrodes will induce opposite charges, forming a current in the external circuit. The PTFE film is then brought back into contact with the PI, while the charges attract one another to form a steady state, and an external circuit neutralizes the graphene electrode's charges. The working mechanism of the single‐electrode TENG is shown in Figure 2b. The contact separation motion of PTFE and an external object causes the induced charge in the graphene electrode to accumulate or dissipate, resulting in the formation of a current in the external circuit. The simulation results of the potential distribution between the two electrodes during the contact separation of the PTFE film and the PI paper are shown in Figure 2c. The simulation result of the single‐electrode TENG is shown in Figure S1 (Supporting Information). To confirm the graphitic structure of LIG, the representative laser‐treated PI paper under 1.25 W laser power (LIG‐1.25) was selected and tested. The laser‐irradiated structures were characterized by scanning electron microscopy (SEM), transmission electron microscopy (TEM), Raman, X‐ray photoelectron spectroscopy (XPS), and energy dispersive spectroscopy (EDS). Firstly, the surface morphology of LIG is revealed by SEM images at low and high magnifications as shown in Figure 2d. It is evident that a foam‐like structure with evenly distributed porous materials has been formed. TEM images (Figure 2e) further reveal the corrugated structure, confirming the ultrathin nature of LIG with the lattice space of ≈3.4 Å. Figure 2f then shows significant D‐peak (≈1350 cm^−1^), G‐peak (≈1592 cm^−1^), and 2D peak (≈2689 cm^−1^) in LIG‐1.25 compared to the featureless Raman shift of the original PI, representing the inherent properties of few‐layered graphene.^[^ ^41^ ^]^ The XRD pattern in the supplementary materials confirms the presence of a multilayer internal structure of graphene, and the strong peaks (002) and (100) are concentrated at 2θ = 26.3 and 2θ = 43.5, respectively (Figure S2d, Supporting Information). After irradiation, XPS spectroscopy (Figure 2g) confirms a significant increase in carbon (from 69.45% to 93.16%) and a decrease in oxygen (from 20.58% to 4.89%) and nitrogen (from 9.97% to 1.95%) elements. Additional information about the elements is shown in Figure S2 (Supporting Information). Figure 2h shows the EDS mapping of LIG, indicating that carbon (C), oxygen (O), and nitrogen (N) are evenly distributed on the surface of the processed PI paper.

### 2.2 Output Performance of the PIP‐TENG

**Figure 3:**
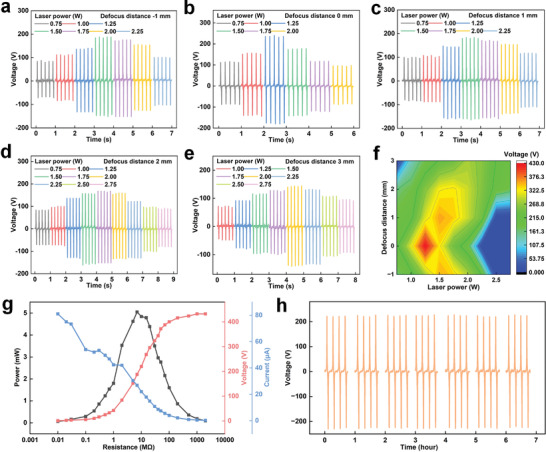
Open‐circuit voltage of PIP‐TENG processed by various laser powers under a specific defocus distance of a) ‐1 mm, b) 0 mm, c) 1 mm, d) 2 mm, and e) 3 mm. f) Open‐circuit voltage distribution tuned by varied laser powers and defocus distances. g) The output voltage, current, and electric power of PIP‐TENG as a function of external resistance. h) Long‐term test of PIP‐TENG performed under 108000 contact‐separation cycles for ≈6 h.

Friction materials and electrodes can directly affect the output performance of TENG, thus the production of graphene electrode layers is essential.^[^ ^51^ ^]^ Therefore, the relationship between laser power, degree of defocus, and TENG performance has been investigated. By setting the excitation frequency of load to 5 Hz, the output performance of PIP‐TENG (60 × 60 mm^2^) processed under various laser power and defocus distance conditions was evaluated by comparing the output open‐circuit voltages as shown in **Figure** **3**a–e. In Figure 3a, the defocus distance of laser is fixed at −1 mm, and the open‐circuit voltage of PIP‐TENG increases initially and then decreases as the laser power is increased from 0.75 to 2.25 W. When the laser power is 1.50 W (LIG‐1.50), the open‐circuit voltage (≈350 V) reaches the highest value. When the defocus distances are 0, 1, 2, and 3 mm, the open‐circuit voltage shows the same trend, by respectively reaching the maximum open‐circuit voltage at 430, 356, 328, and 280 V (Figure 3b–e). To better compare the performance of TENG under different processing parameters, the distribution of open‐circuit voltage is summarized in Figure 3f. With the increase of laser power, the open‐circuit voltage displays a certain distribution at various defocus distances to form a contour structure. When the defocus distance is 0 mm, the peak value of voltage points to the laser power condition of 1.25 W, whereas the peak value points to 1.50 W when the defocus distance is −1 mm or 1 mm. By further increasing the defocus distance to 2 or 3 mm, the voltage peak shifts toward higher laser powers. It is concluded that the greater the loss of focus, the higher power is required to form a porous LIG structure with excellent electrical properties to guarantee the TENG device with superior electric energy ouput performance.

Under the establishment of process‐property relationship, the open‐circuit voltage reaches the maximum value of 430 V (11.9 V cm^−2^) when the laser defocus distance is 0 mm and the laser power is 1.25 W.To further calculate the output performance of electric power, different external resistances have been connected to the external circuit to explore the output voltage, current and power of PIP‐TENG as a function of external resistance. In Figure 3g, as the increase of external resistance from 0.01 to 2000 MΩ, the ouput voltage or current respectively reflects a monotonic increase or decrease. This accordingly promises the performance of output electric power exhibiting a peak value, which is around 5 mW when the external resistance is 7 MΩ. This power level is convinced to drive low‐power devices such as sensors and LEDs. Meanwhile, long‐term durability is crucial to influence the TENG performance, thus a 6‐hour long‐term test of PIP‐TENG was implemented under conditions of 22 ℃ and 10% relative humidity (Figure 3h). After approximately 108,000 contact‐separation cycles, nearly 100% of the original performance (≈430 V) could be maintained. During the test, the surface and cross‐section morphologies of LIG were captured in every 2 h, revealing no apparent change (Figure S5, Supporting Information).

**Figure 4:**
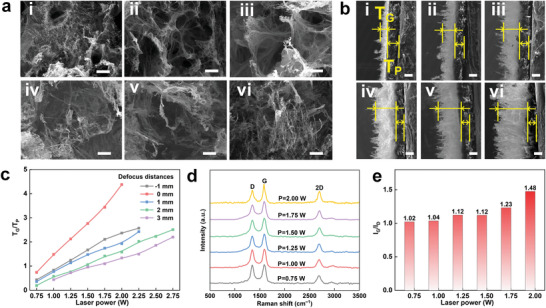
a) SEM images of LIG processed under varied laser powers (scale = 3 µm). b) SEM images of cross‐sectional LIG processed under varied laser powers (scale = 100 µm). c) The ratio of carbonization thickness to residual PI thickness tuned by varied laser powers and defocus distances. d) Raman spectra of LIG processed under different laser powers. e) Intensity ratios of G and D peaks calculated from Raman spectra as a function of laser power.

The above results reveal that the optimal TENG performance could be achieved at specific laser conditions, in which the laser power is one of the key parameters for tuning TENG performance. To further evidence the laser power dependent performance, surface morphology and cross‐sectional structure of the graphene layer have been studied and analyzed as shown in **Figure** **4**a. Considering 0.75 W is the threshold condition to carbonize PI, only partial surface of  the irradiated PI paper exhibits the porous structure(Figure 4a i). When the power increases to 1.00 and 1.25 W, the original PI fiber completely disappears, and the porous structure is much more significant (Figure 4a ii–iii). The dynamic evolution of pore growth, ranging from nearly nonexistent (LIG‐0.75) to a large number (LIG‐1.00) and ultimately to interlaced formations (LIG‐1.25), could be attributed to gas release and graphitization during the irradiation process of laser. Before reaching 1.25 W, higher laser power can increase the porosity and thickness of graphene, as shown in SEM Figure 4a i–iii and Figure 4b i–iii, where the enlargement of graphene medium could serve as an excellent electronic induction layer. For example, both LIG‐1.00 and LIG‐1.25 exhibit uniform 3D porous structures, consequently leading to the continuous enhancement in the performance of TENG. When the laser power exceeds 1.25 W, the thickness of carbonization layer continues to expand, resulting in fewer remnants of the friction layer (PI paper) with thinner thickness, thereby decreasing the performance of TENG (Figure 4b iv–vi). As shown in Figure 4b, the thickness of the carbonization zone is set as *T*_G_, and the thickness of the residual PI is set as *T*_P_. Figure 4c clearly shows that as the increase of laser power, *T*_G_/*T*_P_ reflects a monotonic increase regardless of the applied defocus levels.

In the Supporting Information, local structures (Figures S3–S4, Supporting Information) and cross‐sectional structures (Figures S6–S9, Supporting Information) of other parameters are displayed. The intensity ratio (*I*_G_/*I*_D_) of peak G and peak D determined from the Raman spectroscopy is effective for determining grain size (*L*_a_) on the A‐axis of graphite materials. The comparison of Raman data from 0.75 W to 2 W power is shown in Figure 4d. The *L*_a_ value of the average *I*_G_/*I*_D_ ratio calculated by the formula (1) is shown in Figure 4e. Due to the increase of surface temperature caused by the increase of laser power, the D‐peak gradually decreases, and the *I*_G_/*I*_D_ ratio gradually increases, indicating that the degree of carbonization of PI paper is increasing.^[^ ^52^ ^]^

(1) $$L_{a}=\left(2.4\times 10^{-10}\right)\times \gamma^{4}\times \left(\frac{I_{G}}{I_{D}}\right)$$

*I*_G_ is the integrated intensity of the G peak, *I*_D_ is the integrated intensity of the D peak, and *γ* is the wavelength of the Raman laser (*γ* = 532 nm).

### 2.3 Foldable PIP‐TENG with High‐Output Performance

**Figure 5:**
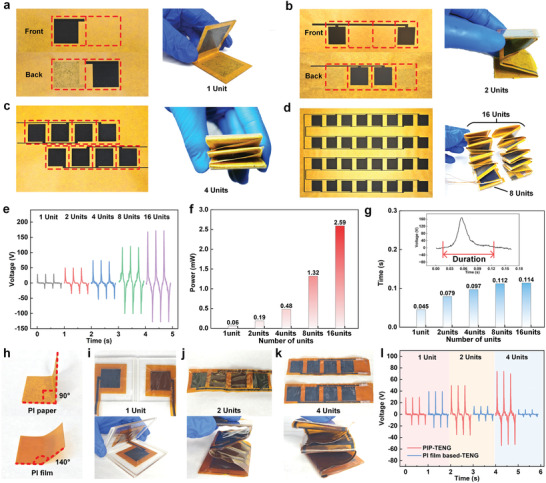
Laser‐irradiated PI paper before and after folded into PIP‐TENG with the friction unit number of a) 1, b) 2, c) 4, and d) 16. e) Comparison of open‐circuit voltage of PIP‐TENG with various friction units. f) Comparison of electric power of PIP‐TENG with various friction units. g) Comparison of voltage‐peak duration of PIP‐TENG with various friction units. h) Comparison of folding states between PI paper and PI film. PI film‐based TENG with i) 1, j) 2, and k) 4 friction units. l) Comparison of voltage performance between PIP‐TENG and PI film‐based TENG.

Although LIG provides a rapid, scalable, patternable, and performance‐optimizable strategy, the performance of PIP‐TENG with single‐unit structure is still limited to the restricted area of friction materials. Ideally, the output performance of PIP‐TENG could be further improved if the total area of friction interface could be increased effectively. Thus, by taking advantage of excellent foldability of PI paper, PIP‐TENG with multi‐unit structures have been newly designed and processed on the basis of optimized irradiation conditions of laser power (1.25 W) and defocus distance (0 mm). Firstly, **Figure** **5**a shows the square LIG pattern processed on both front and back surface of the PI paper before and after folding, in which only one creasing line is needed to fold and assemble the paper material into a single‐unit TENG (30 × 30 mm^2^) with an output performance of ≈48 V. On the basis of single‐unit structure, Figure 5b shows the method to process the PIP‐TENG with two sets of friction units, in which both surfaces require two LIG squares and three creasing lines are needed to fold the device into a zigzag format. Thus, the total friction area can be doubled. To multiply enhance the friction area, Figure 5c shows the method to prepare two PI papers with the total of eight LIG squares and the two papers are cross‐folded together to create the PIP‐TENG with four sets of friction units. Similarly, PIP‐TENG with higher magnitude of friction units can be easily processed by further expanding the number of LIG squares and accumulating the folding layers. For example, a PIP‐TENG with the origami structure including the total of 16 friction units has successfully been assembled by folding and connecting two 8‐unit PIP‐TENG, as shown in Figure 5d. With the superposition of multiple friction units to effectively enhance the total friction area, Figure 5e indeed proves the enhancement of power generation performance. Specifically, as the enlargement of friction unit from 1 to 16, the open‐circuit voltage has dramatically increased from 48 V (equivalent to 5.3 V cm^−2^) to 308 V (equivalent to 34.4 V cm^−2^). Figure 5f further compares the output electric power of each prototype, in which the power increases over 40 times from 0.06 to 2.59 mW as the increase of the unit number from 1 to 16. It is also noticed that the output performance is not increased linearly as the unit number. This could be caused by the loss of some portion of effective friction area under the complicated origami structure, and the potential induction of each unit cannot be fully synchronized during the loading process. Due to the increased thickness of device as the accumulation of folding layers, the contact time among electrodes could also be lengthened during reciprocating motion. Figure 5g confirms that the duration of voltage peak is clearly enlarged from 0.045 to 0.114 s when the unit number of device increases from 1 to 16. Finally, to prove the unique advantage of PI paper for assembling the structure‐foldable PIP‐TENG, two types of raw materials, i.e. PI film (Kapton) and PI paper, have been compared in terms of folding capability, device assembling and output performance. Firstly, both PI film and PI paper are cut to the same size and folded completely. After folding, Figure 5h shows clearly that the PI paper can maintain an angle of about 90° which is similar to cellulose papers, while the PI film gradually recovers to 140°. The above experiment demonstrates that PI paper exhibits superior foldability and can form complex 3D structures. Accordingly, the PI film is almost not able to produce TENG with multiple friction units through folding, as shown in Figure 5i–k. Certainly, the performance of PI film based TENG is worse than that of PIP‐TENG, especially when unit number is increased (Figure 5l). For example, unlike the performance enhancement of PIP‐TENG, the performance of PI film based TENG with 2 or 4 units rapidly decreases from 60 V (1 unit) to 25 V or 13 V (Figure 5l).

### 2.4 Applications of Structure‐Foldable PIP‐TENG

**Figure 6:**
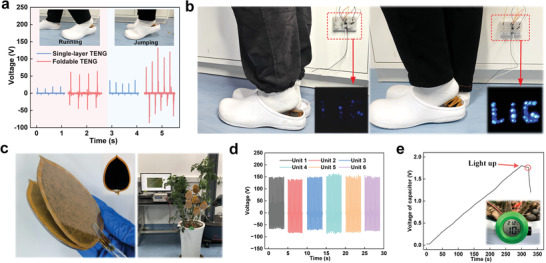
Applications of PIP‐TENG in energy harvesting. a) Movement monitoring implemented by single‐unit and foldable PIP‐TENG. b) Comparison of lightened LED between energy supply from single‐unit PIP‐TENG and from foldable PIP‐TENG. c) Assembly and installation of leaf‐shaped PIP‐TENG. d) Voltage performance of leaf‐shaped PIP‐TENG with various number of friction units and e) charging curve of the PIP‐TENG for powering a thermometer.

To demonstrate the processing, adaptability, and functionalization benefits of PIP‐TENG, prototypes for diversified applications have been developed. First, the PIP‐TENG can be used to collect the energy of human walking and jumping, as well as to detect other information about human gait. **Figure** **6**a shows the electrical signals of the origami structure monitoring human running and jumping, and the magnitude and frequency of the voltage reflect human foot behavior. Under the same action, origami structures collect more energy and provide stronger feedback signals than single‐unit structures. Simultaneously, the foldable PIP‐TENG collects the kinetic energy of the feet to drive 22 LED lights, whereas a single‐unit TENG structure lacks the capability to drive these LEDs (Figure 6b; Movie S1, Supporting Information). Additionally, the PIP‐TENG is formed into the shape of a leaf and attached to a tree to harvest natural environmental energy. The individual performance of the leaf‐shaped TENG is ≈200 V (Figure 6c–d). Six units in parallel can successfully drive the temperature and humidity sensor (Figure 6e) or 60 LED lights (Movies [Link], [Link], Supporting Information) under simulated wind conditions (≈6 m s^−1^). The above experiments have proven that foldable TENG has good energy‐harvesting effects.

**Figure 7:**
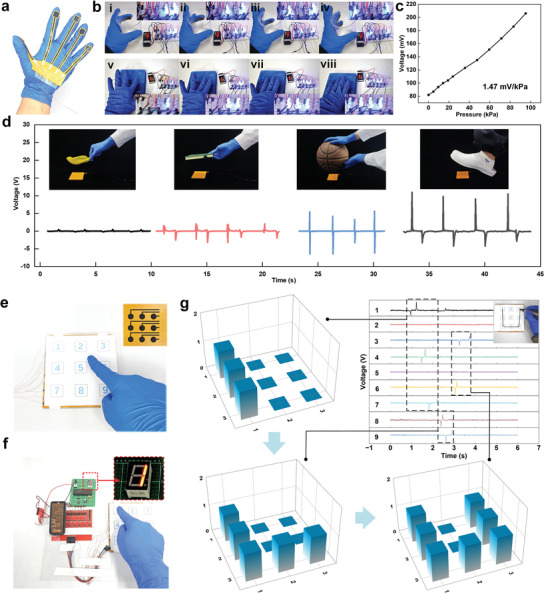
Demonstrations of PIP‐TENG in smart sensing. a) Picture of a palm tactile sensor. b) Touch sensing from varied fingers instructed by LED lightning. c) The linear correlation between pressure and voltage from the tactile sensor. d) Recognition of objects by the tactile sensor. e) Picture of a matrix sensor. f) Screen control by using the matrix sensor. g) Recognition of writing gestures by the matrix sensor through different electrical signals.

The above experiments have demonstrated that the TENG processed by LIG technology can effectively convert energy from the environment and motion into electricity. Additionally, laser processing can customize the shape of sensors. This study introduces a palm tactile sensor. The sensor converts the touch signal of five fingers into the flashing signal of the LED light using the transistor (**Figure** **7**a–b). When the corresponding finger touches the beaker, the sensor output voltage will activate the transistor, and the corresponding LED light will be driven (Figure 7b i–iv). Different numbers of fingers are pressed on the panel, and different series of LEDs are driven together for a while (Figure 7b v–viii; Movie S4, Supporting Information). Figure S10 (Supporting Information) demonstrates that the palm sensor generates two, three, four, and five‐finger signals when the user grasps the pen, box, keyboard, and beaker, respectively. The peak and time domain of the signal can be used to determine the force and velocity of the hand movements. Furthermore, a pressure test ranging from 0 to 95 kPa was designed to demonstrate the pressure‐sensing characteristics of the tactile sensor (10 × 10 mm^2^). The tactile sensor exhibits excellent pressure‐sensing properties (Figure 7c). By receiving voltage signals, the PIP‐TENG can automatically recognize contact objects such as feathers, notebooks, basketballs, and foot movements through learning and training (Figure 7d; Movie S5, Supporting Information). Laser selection processing was further utilized to fabricate matrix sensors (Figure 7e). The voltage of each touch unit is essentially the same (≈2 V, Figure S11, Supporting Information), and the matrix sensor can not only control the digital display in conjunction with intelligent circuits (Figure 7f) but also recognize writing gestures via electrical signals (Figure 7g) to remote text input. To sum up, the above experiments demonstrate the structural adaptability and application diversity of the PIP‐TENG, which could have broad future application prospects in smart skin, intelligent sensing, natural energy capture, soft robots, the Internet of Things, and etc.

## 3 Conclusion

It can be concluded that the PI paper‐enabled PIP‐TENG technology can greatly increase production efficiency. A series of PIP‐TENG with foldable structure and tailorable performance have been produced by precise control of critical laser parameters. At optimal laser parameters, the open‐circuit voltage of PIP‐TENG can reach 430 V (11.9 V cm^−2^), with a peak power of 5 mW. The origami TENG with a plane area of 30 × 30 mm^2^ can dramatically enhance its output electric power for over 40 times, while the open‐circuit voltage has effectively been increased from 5.3 to 34.4 V cm^−2^. Representative e nergy‐harvesting and smart‐sensing applications have been demonstrated, including a smart shoe, a smart leaf, a matrix sensor, and a smart glove. The smart shoe and smart leaf can harvest human kinetic energy and wind energy to power temperature‐ and humidity‐sensing devices and LEDs. Additionally, the smart glove could accurately monitor finger pressure and touch, and the matrix sensor was developed as an intelligent input to recognize writing trajectories. Overall, the proposed PIP‐TENG technology is highly expected to promote the development and broaden the application scenarios of TENG in environmental energy harvesting, smart sensors and intelligent robots in the future.

## 4 Experimental Section

### Fabrication of the PIP‐TENG

A laser platform (DLS 2.3, Universal Laser Systems, Inc.) equipped with a CO_2_ laser with a wavelength of 10.6 µm and beam size of ≈100 µm was used for irradiating PI paper (Cat.# Yilun‐P‐90, PolyKing Co., 90 µm in thickness). Varied laser powers (0.5–2.75 W) with increments of 0.25 W were used to scribe line‐by‐line on both sides of PI with the overall size of 10 × 10 mm^2^, 30 × 30 mm^2^, and 60 × 60 mm^2^. The process was performed under ambient. The negative friction material is PTFE with a thickness of 80 µm. The outer protective layer of the electrode is nylon with a thickness of 50 µm. The backing material is acrylic.

### Electrical Measurement

The excitation platform was a linear motor (B01‐37 × 120/160, LinMot). The voltage of the PIP‐TENG is measured by a spectrum analyzer (MDO3034, Tektronix, USA), and the capacitance–voltage was tested by a digital multimeter (7510, Keithley, USA). The pressure sensing range of TENG was evaluated using an E44.104 tensile machine (10 kN load cell, MTS Systems Corp.). SEM was performed with JEOL JSM 7001F at 10 kV to examine the morphologies of the laser‐scribed features. A Horiba HR800 Raman microscope was employed to obtain Raman spectra with a 532 nm excitation laser at the power of 5 mW. XRD characterization was performed on Rigaku D/max 2550 with Cu Kα radiation (*λ* = 1.54 Å). XPS (Thermo Fisher ESCALAB 250Xi) was applied to compare the content of surface elements. Prepared by dropping of LIG‐alcohol suspension on a Cu grid, TEM images were taken using an 80 KeV JEM 2100F (JEOL Inc.) for evaluation of LIG at nano scale.

All volunteers have known all the details about the experiment. The experiment results will be used to conduct further research. Beihang University will ensure the health and safety of all volunteers in the experiment. All volunteers have agreed to participate in the experiment.

## Conflict of Interest

The authors declare no conflict of interest.

## Supporting information

Supporting Information

Supplemental Movie 1

Supplemental Movie 2

Supplemental Movie 3

Supplemental Movie 4

Supplemental Movie 5

## Data Availability

The data that support the findings of this study are available from the corresponding author upon reasonable request.
