# Supplementary material for: Structure‐Foldable and Performance‐Tailorable PI Paper‐Based Triboelectric Nanogenerators Processed and Controlled by Laser‐Induced Graphene
Source: Adv Sci (Weinh). 2024 May 15;11(28):2310017. doi: 10.1002/advs.202310017 (PMC11267377; doi:10.1002/advs.202310017)
Supplement: Supplementary file 1 — Supporting Information [file ADVS-11-2310017-s001.pdf]

ADVANCED SCIENCE
Open Access

Supporting Information

for *Adv. Sci.*, DOI 10.1002/advs.202310017

Structure-Foldable and Performance-Tailorable PI Paper-Based Triboelectric Nanogenerators Processed and Controlled by Laser-Induced Graphene

*Weixiong Yang, Mingguang Han, Fu Liu, Dan Wang, Yan Gao, Guantao Wang, Xilun Ding and Sida Luo**

Supporting Information

Structure-foldable and performance-tailorable PI paper-based triboelectric nanogenerators processed and controlled by laser-induced graphene

Weixiong Yang, Mingguang Han, Fu Liu, Dan Wang, Yan Gao, Guantao Wang, Xilun Ding, Sida Luo *

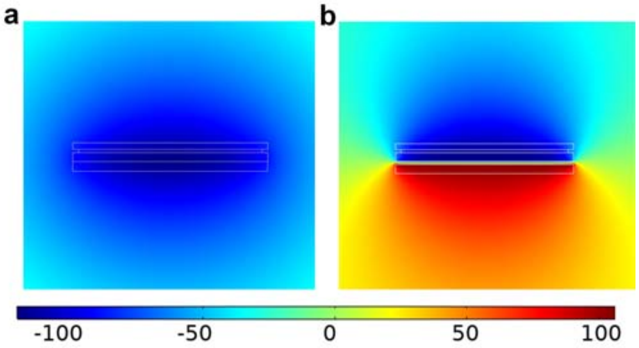

Figure S1. The simulation diagram of the single-electrode TENG.

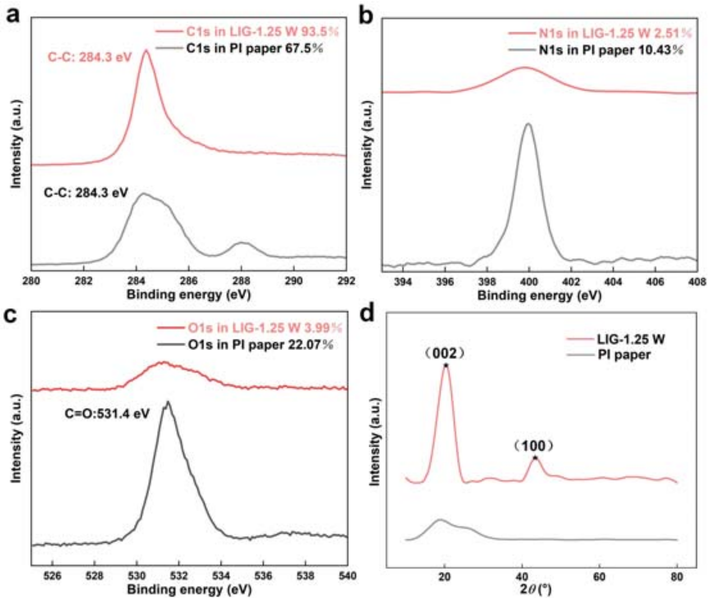

Figure S2. XPS pattern of C1s (a), O1s (b) and N1s (c) of the original PI and LIG-1.25 (d) XRD pattern of LIG-1.25 and the original pi paper.

High resolution C1s XPS spectrum of the LIG film and PI, showing the dominant C—C peak. The C—N, C—O and C=O peaks from PI were greatly reduced in the C1s XPS spectrum of LIG, which indicates that LIG was primarily sp^2- carbons

(Figure S2a). High resolution N1s XPS spectrum of a LIG-1.25 W film and PI. The intensity of the N1s peak was greatly reduced after laser exposure (Figure S2b). High resolution O1s XPS spectrum of a LIG-1.25 W film and PI. After laser conversion, the C—O (531.2 eV) peak becomes more dominant than C=O (531.4 eV) (Figure S2c). The XRD pattern in the supporting information confirmed the existence of multilayer internal structure of graphene, and the strong peaks (002) and (100) were concentrated at $2\theta = 26.3°$ and $2\theta = 43.5°$, respectively (Figure S2d).

At each irradiation distance, the power parameters with the best voltage performance are 1.5, 1.25, 1.5, 1.75 and 2 W, respectively. Under these parameters, homogeneous porous layers are formed (Figure S3).

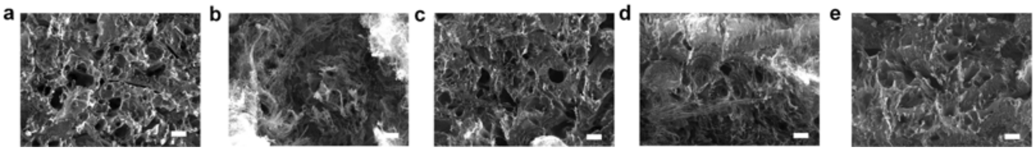

Figure S3. The SEM of maximum voltage at the different defocus distances (-1, 0, 1, 2 and 3 mm).

The SEM images with different irradiation distances at 1.25 W power. Only at defocus distance 0 mm, a porous graphene layer is formed (Figure S4).

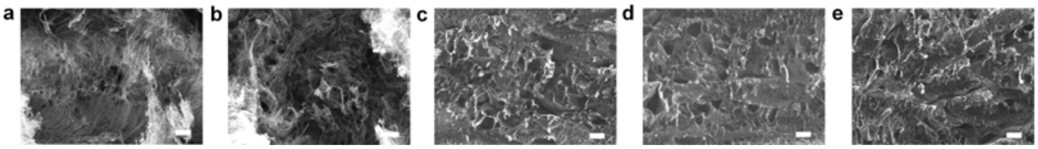

Figure S4. The comparison of different defocus distances (-1, 0, 1, 2 and 3 mm) at the 1.25 W.

Resistance changes at 0, 2, 4, and 6 hours under the durability test (Figure S5). At the same time, SEM images and interface images of different times were also displayed, and after testing with different cycles, the graphene electrode remained basically intact (Figure S6).

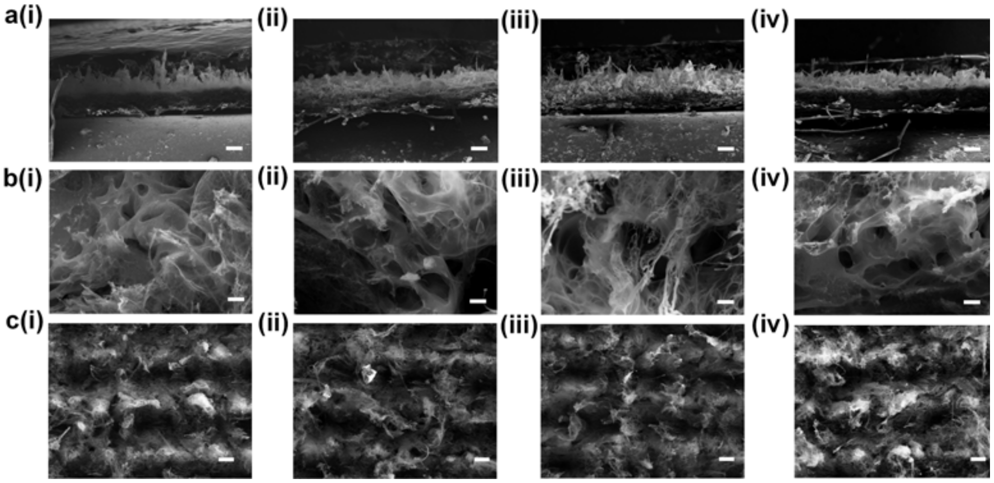

Figure S5. The SEM images under the endurance test: (a) Low (scale = 100 μm) magnified cross-sectional SEM images at 0, 2, 4, and 6 hours. (b) High (scale = 5 μm) magnified SEM images at 0, 2, 4, and 6 hours. (c) Low (scale = 50 μm) magnified SEM images at 0, 2, 4, and 6 hours.

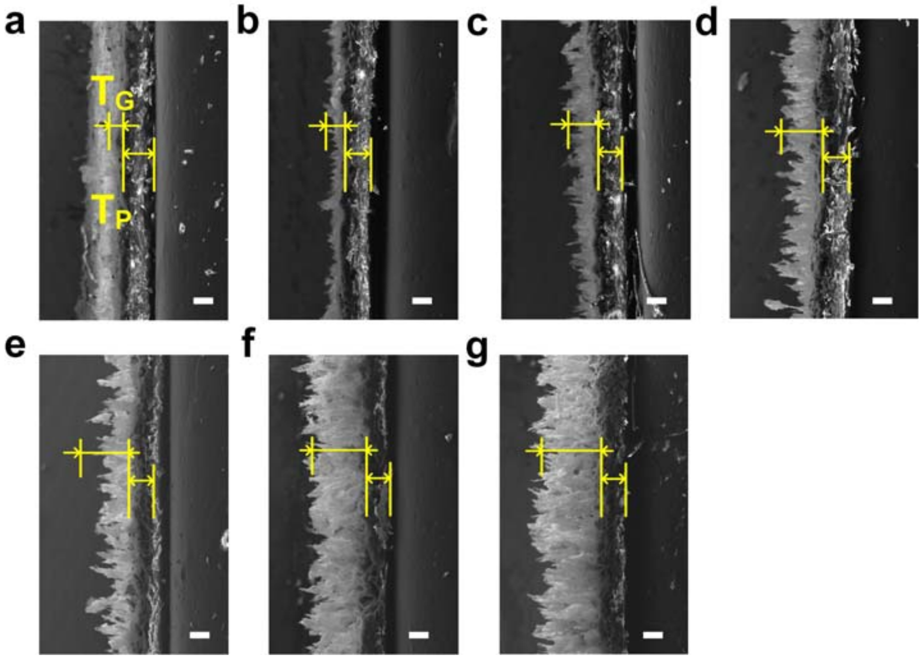

Figure S6. SEM image of cross-section structure when the defocus distance is -1 mm and the power is 0.75 W, 1 W, 1.25 W, 1.5 W, 1.75 W, 2 W, 2.25 W.

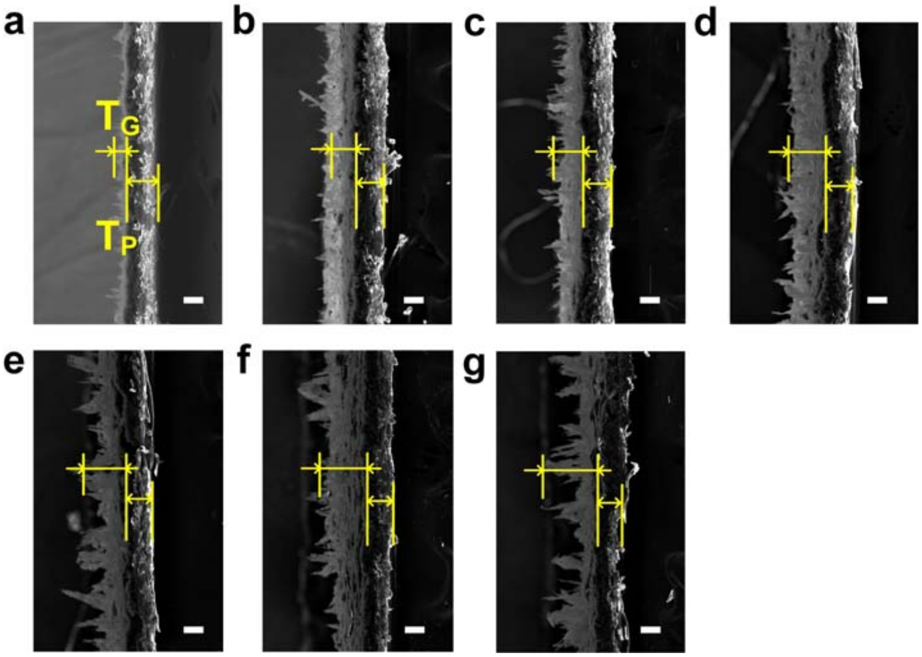

Figure S7. SEM image of cross-section structure when the defocus distance is 1 mm, and the power is 0.75 W, 1 W, 1.25 W, 1.5 W, 1.75 W, 2 W, 2.25 W.

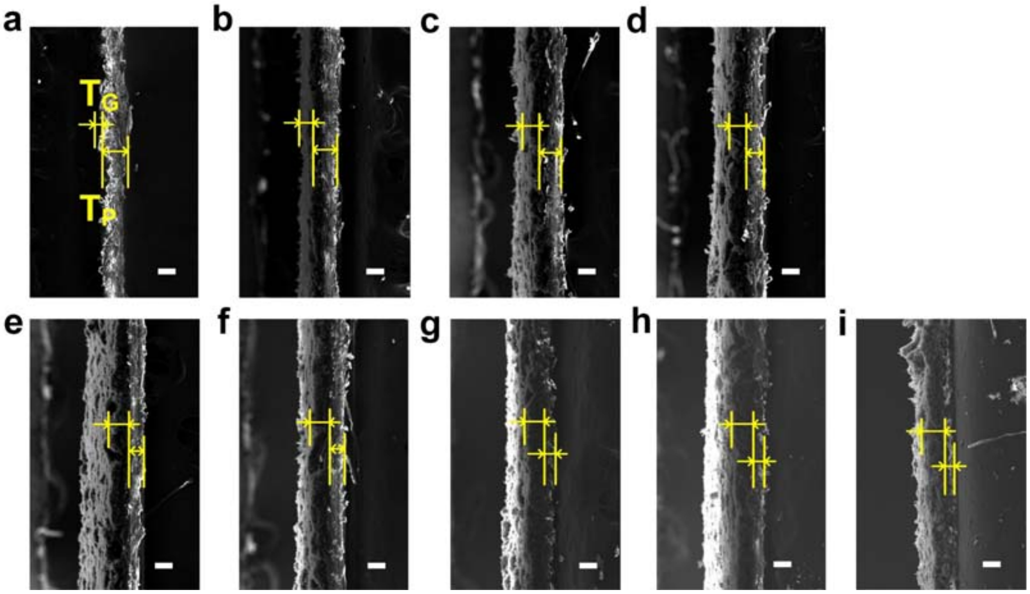

Figure S8. SEM image of cross-section structure when the defocus distance is 2 mm, and the power is 0.75 W, 1 W, 1.25 W, 1.5 W, 1.75 W, 2 W, 2.25 W, 2.5 W, 2.75 W.

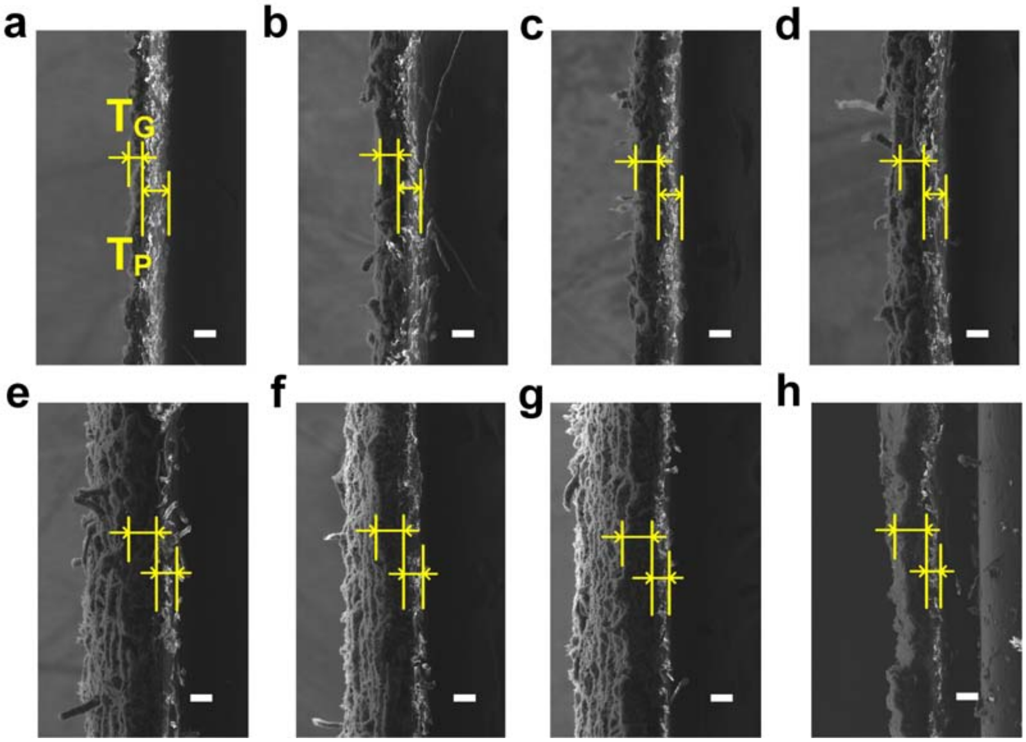

Figure S9. SEM image of cross-section structure when the defocus distance is 3 mm, and the power is 1 W, 1.25 W, 1.5 W, 1.75 W, 2 W, 2.25 W,2.5 W, 2.75 W.

The laser induced graphene technology produces a tactile sensing system. The tactile sensor generated two, three, four and five finger signals when grasping the pen, box, keyboard and beaker, respectively. (Figure S10a-d). The performance of each unit of the matrix sensor is basically consistent, approximately 2 V. At the same time, the matrix sensor monitored the hand movements of writing BUAA.

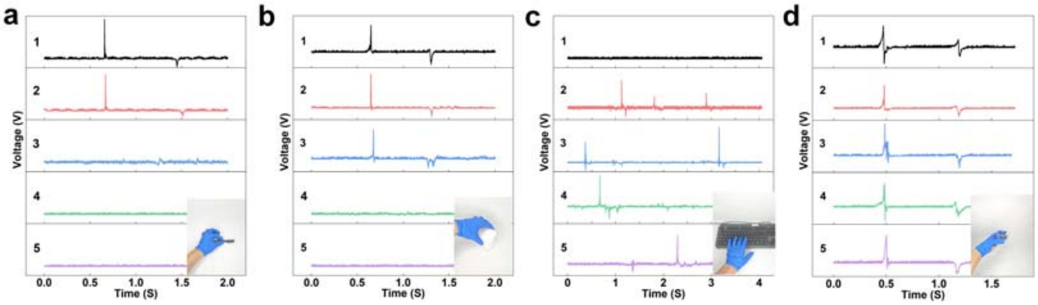

Figure S10. Demonstration of the PIP-TENG in smart sensing. (a-d) The palm sensor generated two, three, four and five finger signals.

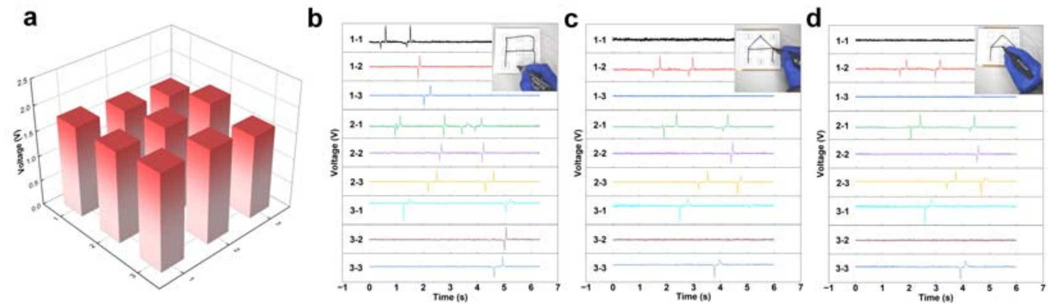

Figure S11. Demonstration of the PIP-TENG in smart sensing. (a) The voltage of each touch unit is basically the same (about 2 V). (b-d) Matrix sensors monitor writing movement.

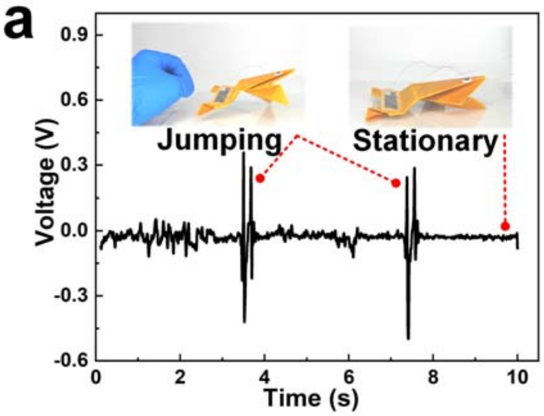

Figure S12. Sensors recognize signals from origami frog jumps.

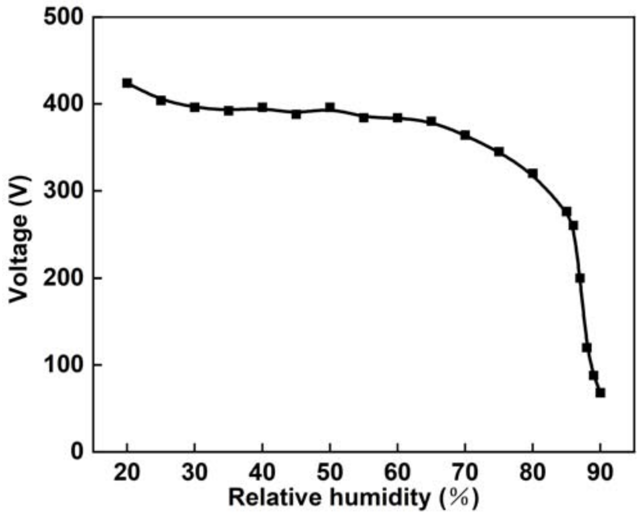

Figure S13. TENG performance at different relative humidity levels.

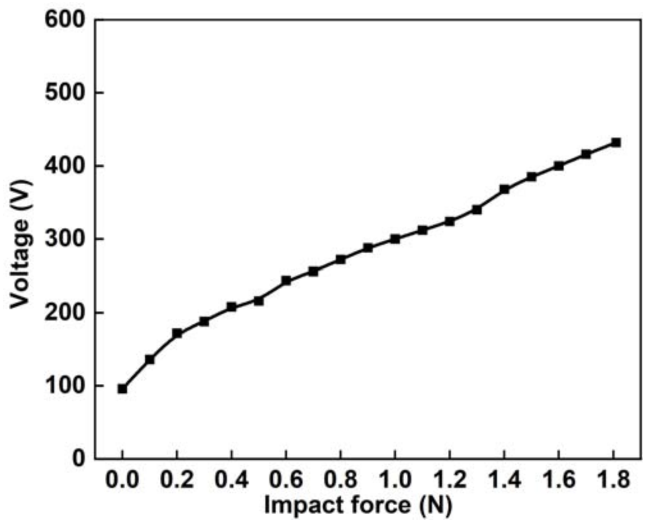

Figure S14. The relationship between impact force and TENG performance.

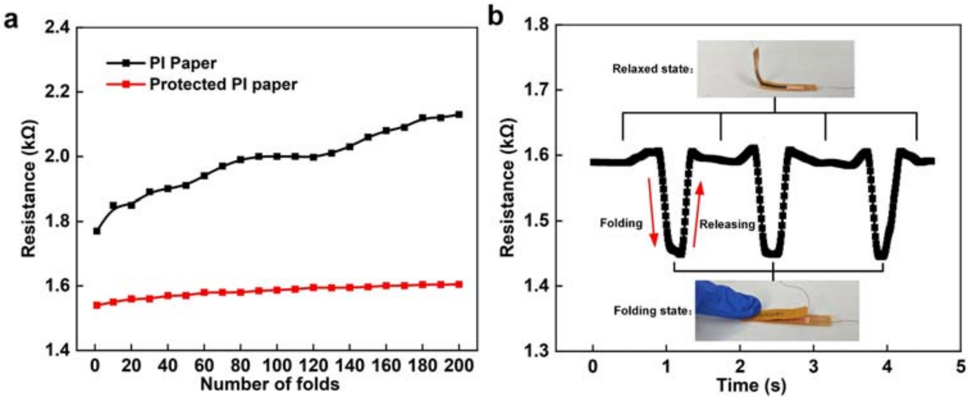

Figure S15. (a) Resistance of LIG electrodes under different folding times. (b) The variation curve of resistance during the folding process.

0
